# Determination of the Structure of the Catabolic *N-*Succinylornithine Transaminase (AstC) from *Escherichia coli*

**DOI:** 10.1371/journal.pone.0058298

**Published:** 2013-03-06

**Authors:** Janet Newman, Shane Seabrook, Regina Surjadi, Charlotte C. Williams, Del Lucent, Matthew Wilding, Colin Scott, Thomas S. Peat

**Affiliations:** 1 CSIRO Materials, Science and Engineering, Parkville, Australia; 2 CSIRO Ecosystem Sciences, Canberra, Australia; University of Canterbury, New Zealand

## Abstract

*Escherichia coli* possesses two acyl ornithine aminotransferases, one catabolic (AstC) and the other anabolic (ArgD), that participate in L-arginine metabolism. Although only 58% identical, the enzymes have been shown to be functionally interchangeable. Here we have purified AstC and have obtained X-ray crystal structures of apo and holo-AstC and of the enzyme complexed with its physiological substrate, succinylornithine. We compare the structures obtained in this study with those of ArgD from *Salmonella typhimurium* obtained elsewhere, finding several notable differences. Docking studies were used to explore the docking modes of several substrates (ornithine, succinylornithine and acetylornithine) and the co-substrate glutamate/α-ketogluterate. The docking studies support our observations that AstC has a strong preference for acylated ornithine species over ornithine itself, and suggest that the increase in specificity associated with acylation is caused by steric and desolvation effects rather than specific interactions between the substrate and enzyme.

## Introduction

The catabolism of L-arginine by bacteria has been an area of extensive study. In part, these studies have been driven by the role of L-arginine catabolism in various potential applications. For example, disruption of L-arginine catabolism can lead to strains that over-produce L-arginine and may be useful for the multi-billion dollar amino acid production industry [1] and L-arginine can be used as a feedstock for the synthesis of polyamines such as 1,4-diaminobutane [2] and putrescine [3], which have utility in polymer production. Enzymes used in the catabolism of arginine, and related enzymes, have other potential biotechnological applications, particularly in the development of efficient routes for the production of chiral amines, used in the production of pharmaceutical products [4].

There are four well characterized pathways for L-arginine degradation: the arginase pathway [5], [6], the arginine deiminase pathway [7]–[9], the arginine transaminase pathway [10]–[12] and the arginine-succinyltransferase pathway [13]. Some organisms, such as *Pseudomonas aeruginasa,* possess several pathways (i.e. arginine-succinyltransferase, arginine deiminase and arginine transaminase pathways) [14]. In contrast, it is also common for organisms to possess a solitary pathway, e.g. *Bacillus subtilis* has only the arginase pathway [15] and *Escherichia coli* has only the arginine-succinyl transferase pathway [16].

**Figure 1:**
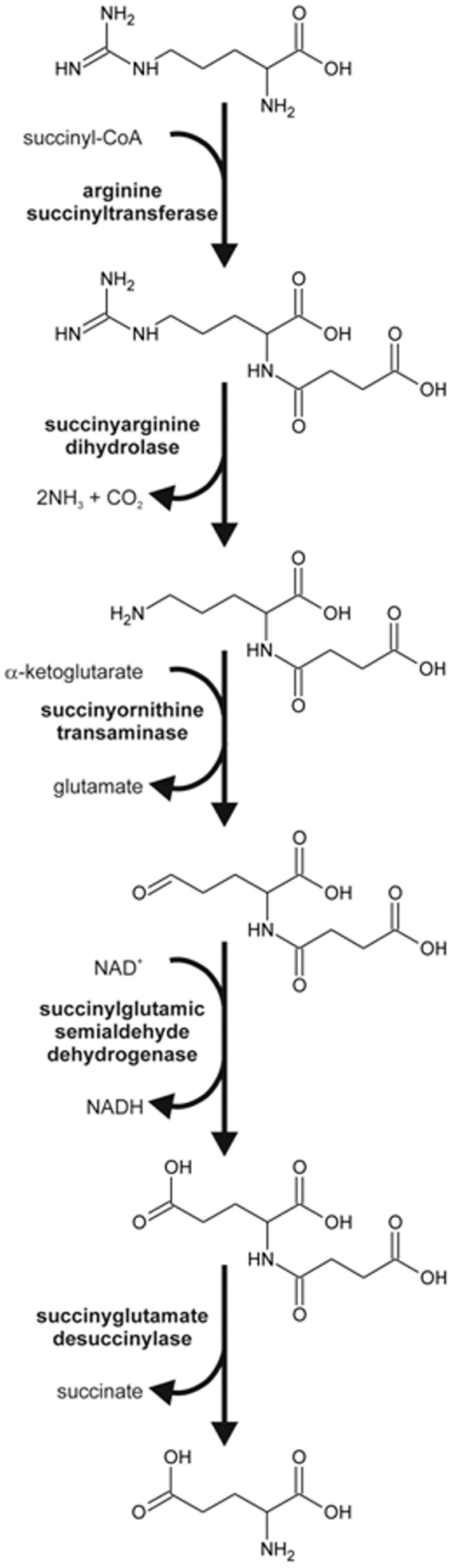
The Arginine degradation pathway.

The arginine-succinyl transferase pathway of *E. coli* provides nitrogen for growth *via* a series of succinylated intermediates (Fig. 1) [16]. The pathway is encoded by the *astCADBE* operon, which is highly regulated in response to nitrogen and general starvation through the coordinated actions of the transcription factors RpoN, RpoS, NtrC, CRP and ArgR [17].

The first gene of the arginine catabolism operon, *astC (*also called *cstC, argM* or *ydjW)*, encodes one of two acetyl/succinylornithine aminotransferases (SOAT, EC 2.6.1.11/2.6.1.81) present in *E. coli* [18]. AstC is a dimeric, pyridoxal phosphate (PLP)-dependent δ–aminotransferase that operates a ping-pong bi-bi reaction mechanism with succinylornithine (SO) and α-ketoglutarate (αKG) as co-substrates and succinylglutamic semialdehyde and glutamate as co-products [18] (Fig. 1).

The second *E. coli* SOAT is the anabolic ArgD, which is 58.6% identical to AstC. ArgD is required for the biosynthesis of arginine *via* a series of *N-*acetyl intermediates, including *N-*acetylornithine. In 1998, Ledwidge and Blanchard showed that ArgD is identical to the long-sought *dapC* gene product (DapATase) which catalyzes the glutamate-dependent transamination of *N*-succinyl-L-2-amino-6-oxopimelate to generate *N*-succinyl-L,L-diaminopimelate, required for the biosynthesis of lysine [19]. X-ray structures for ArgD from a number of different bacteria including *Salmonella typhimurium* (e.g. PDB code 2PB2 and 2PB0), *Thermus thermophilus* (PDB code 1VEF), *Aquifex aeolicus* VF5 (PDB code 2EH6), *Campylobacter jejuni* (PDB code 3NX3) and *Thermotoga maritima* (PDB code 2ORD) have been determined, demonstrating that this enzyme is also a classical dimeric PLP-dependent, αβ-fold protein.

ArgD from *E. coli* and *S. typhimurium* have been characterized biochemically and shown to have *k*_cat_ values of 0.22–0.82 s^−1^ and 0.61–1.6 s^−1^ for ornithine (ORN) and acetylornithine (AO), respectively, and *K*_M_ values of 4,500-640 µM and 150-37 µM for ORN and AO, respectively [19], [20] . The equilibrium constant (*K*_eq_) for this reaction is close to 1, suggesting that the enzyme can operate efficiently in either direction depending upon the relative concentrations of substrate and product. Indeed, AstC expressed from a plasmid is able to complement an *argD* deletion [16] [21].

Here we describe the structural and biochemical characterization of the catabolic SOAT from *E. coli*, AstC, finding differences from the structures of the related anabolic SOAT enzyme (ArgD). We also propose a plausible explanation for the greater specificity of AstC for AO compared with ORN itself.

## Materials and Methods

### Purification of AstC

The native, untagged form (i.e. no plasmid or clone used) of AstC from *E. coli* (BL21 DE3) was purified from a bacterial growth in a rich medium (2xYT) using affinity (Ni-NTA) and size exclusion chromatography. Although AstC is not one of the 10 *E. coli* proteins found to bind tightly to Ni-NTA columns by Tiwari *et al*, it is likely that under the growth conditions used in their study AstC was not highly expressed [22]. We estimate the purity of the protein after these two steps to be >95% (from Coomassie stained SDS PAGE analysis, data not shown). The protein was subsequently concentrated in a 10 kDa Amicon spin concentrator to approximately 5 mg/mL for crystallization trials. The concentrated protein solution was clear and colorless. Holo-enzyme was obtained by adding an excess of PLP (∼10 fold) to the apo-enzyme and incubating this on ice for several hours.

### Crystallography

A long, needle-like crystal of the PLP-free enzyme grew from a reservoir containing 1.4 M sodium malonate (pH 7), 10% (v/v) malate-MES-Tris buffer [23] pH 5 at 20° C in droplets set up in a ratio of 50∶25:25 protein:reservoir:silver bullet #62 (HR2-996-62, Hampton Research, USA) with a total volume of 400 nL – see Figure S1-A. The Schiff base-enzyme complex (PLP bound) crystals were grown at 8° C in 1.17 M sodium malonate (pH 7), 0.09 M Tris chloride pH 8.0, 0.02 M di-ethylammonium formate in drops that were 300 nL initial volume, consisting of equal volumes of protein and reservoir – see Figure S1-B. The crystals with PLP bound in the protein, but the Schiff base broken were grown under similar conditions to those with the Schiff base intact, but ORN as a dry solid was added to the crystallization drop 2 days prior to harvesting the crystals and collecting diffraction data. This same procedure was used to add αKG to holo-AstC crystals. 10 mM glutamic acid in crystallization cryo-solution (2.2 M sodium malonate pH 7) was added to crystals 3 days before data collection. Co-crystals of AstC in the presence of PLP and SO were grown at 20° C as a 50∶50 ratio of the protein complex with 1-4-1.6 M ammonium sulfate, and pH 7–9 (300 nL initial drop size), see Figure S1-C. All of the crystallizations were set up in SD-2 (IDEX Corp, USA) 96-well sitting drop plates using a Phoenix robot (Art Robbins Industries, USA). Crystals grown with the PLP-enzyme complex were chunkier and grew more reproducibly than the crystals of the apo enzyme.

All X-ray data were collected at the MX-2 beamline of the Australian Synchrotron (see Table 1). The non-PLP crystals adopted the R32:h rhombohedral spacegroup, whereas all crystals grown with added PLP were found the C2 spacegroup. There were 2 molecules in the asymmetric unit of the H32 crystals and 4 molecules in the asymmetric unit of the C2 crystals. Multiple data sets were collected for almost all soaks and co-crystals, and generally 360 degrees of data were collected in 0.5 degree oscillation increments for a total of 720 frames at 13,000 eV X-ray energy. The crystals grown in sodium malonate had no additional cryo-protectant added and were flash-cooled directly in the nitrogen cryo-stream. Crystals grown in ammonium sulfate were cryo-protected by adding 1.5 µL of 2.5 M sodium malonate to the crystallization drop before harvesting the crystals and cryo-cooling in the cold nitrogen stream. The data were indexed using XDS [24] and scaled using SCALA [25](CCP4 software suite [26]). The initial non-PLP bound structure was solved by molecular replacement using Phaser [27] with PDB 2PB2. Subsequent structures were solved using the A protomer from the non-PLP bound structure as the starting model by molecular replacement with Phaser. All structures were built using Coot [28] and refined with Refmac [29].

**Table 1:** Data and model statistics for each of the AstC structures solved.

	APO	PLP	PLP+ SO	PLP+ ORN
**Space group**	R32:h	C2	C2	C2
**Cell (Å)**	231.5×231.5×110.4γ = 120	184.7×118.4×109.8ß = 96.7	183.9×118.3×109.2ß = 96.8	184.4×118.3×109.5ß = 96.8
**Resolution (Å)**	19.8–2.75	19.8–2.20	19.8–2.45	19.8–2.30
**Completeness (%) (high res bin)**	99.3 (99.2)	99.7 (98.0)	99.8 (100)	97.8 (86.6)
**Rwork (%)**	16.8	17.9	17.0	17.8
**Rfree (%)**	21.1	21.8	22.5	20.9
**X-ray source**	MX-2	MX-2	MX-2	MX-2
**Detector**	ADSC 315	ADSC 315	ADSC 315	ADSC 315
**Wavelength ( Å)**	0.9537	0.9537	0.9537	0.9537
**Unique reflections**	29,220	118,526	85,143	101,215
**Observed reflections**	645,137	897,990	502,881	352,029
**Solvent content (%)**	65.3	64.6	64.2	64.3
**Data redundancy (high res bin)**	22.1 (22.4)	7.6 (7.0)	5.9 (5.9)	3.5 (2.7)
**Mean I/σ(I) (high res bin)**	21.9 (6.0)	14.6 (3.4)	10.6 (2.9)	10.7 (3.0)
**Rmerge (high res bin)**	0.150 (0.717)	0.133 (0.610)	0.154 (0.627)	0.098 (0.335)
**Rpim (high res bin)**	0.032 (0.154)	0.052 (0.247)	0.068 (0.279)	0.061 (0.237)
**Size of Rfree set (%)**	5	5	5	5
**Total atoms**	5,775	13,095	12,731	12,925
**water molecules**	30	644	321	518
**Hetero atoms**	0	66	124	63
**RMSD Bond lengths (Å)**	0.020	0.027	0.018	0.026
**RMSD Bond angles (°)**	2.017	2.280	2.097	2.267
**Ramachandran**				
**Favored region (%)**	91.6	94.6	92.2	93.8
**Allowed regions (%)**	6.6	4.9	6.7	5.6
**Disallowed region (%)**	1.8	0.5	1.1	0.7
**Mean B factors (Å^2^)**	33.8	25.2	28.2	22.7

Values in parenthesis are for the high resolution shell.

### Synthesis of Succinylornithine

To a solution of Boc-L-Orn-OH (200 mg, 0.86 mmol) in a 1∶1 mixture of water and dimethylformamide (8 mL) was added triethylamine (480 µL, 3.44 mmol) and succinic anhydride (103 mg, 1.03 mmol). The colorless solution was left to stir at room temperature under nitrogen atmosphere for 14 h and was concentrated under reduced pressure to give a white colored solid. This material was subjected to flash chromatography (silica, 5∶95 acetic acid: 10 % methanol in dichloromethane) to yield Boc protected SO as a white colored foam (246 mg, 86%).

δ_H_ (400 MHz, MeOD) 4.25 (m, 1H, C*H*NH_2_), 3.04 (t, *J* 6.6 Hz, 2H, C*H*_2_-NHBoc), 2.53 (m, 4H, NHCO-C*H*_2_C*H*_2_), 1.84 (m, 1H, C*H*H), 1.66 (m, 1H, CH*H*), 1.52 (m, 2H, C*H*_2_), 1.42 (s, 9H,-*t*-Bu). *m/z* (ESI) calc.: 332.16. Found 355.5 (M+Na+), 687.4 (2 M+Na+).

Boc protected SO (246 mg, 0.74 mmol) was dissolved in anhydrous dichloromethane (2 mL) and trifluoroacetic acid (0.4 mL) was added drop wise; after stirring for 3 h, the mixture was concentrated under reduced pressure. The residue was taken up in 1∶1 acetonitrile:water and the volatiles removed under vacuum. This was repeated twice to ensure complete removal of TFA, to yield SO as a white colored foam (quantitative yield).

δ_H_ (400 MHz, MeOD) 4.28 (m, 1H, C*H*NH_2_), 2.86 (m, 2H, C*H*_2_NH_2_), 2.60 – 2.34 (m, 4H, NHCO-C*H*_2_C*H*_2_), 1.91 (m, 1H, C*H*H), 1.75 – 1.59 (m, 3H, CH*H* and C*H*_2_). See Figure S2 for a schematic representation of the reaction.

### Biochemical Assays

Transaminase activity was measured by glutamate dehydrogenase (GDH) assay [30], [31]. Briefly, the rate of glutamate formation *via* the amination of αKG was monitored by the inclusion of glutamate dehydrogenase and NAD^+^ in the reaction, effectively recycling the αKG and reducing one molecule of NAD^+^ to NADH per glutamate formed. The concentration of NADH was monitored at 340 nm.

Reactions were conducted in 10 mM Tris-HCl (pH 8.0) containing 200 µM αKG, 500 µM NAD^+^, 5 U GDH and 0–2,000 µM ORN, SO, AO. The reactions were carried out in 100 µL volumes in a 96-well, UV-transparent microtiter plate and the change of absorbance at 340 nm was followed using a SpectroMax 190 spectrophotometer reading each well once every 10 seconds. Concentrations of αKG above 200 µM inhibited GDH activity (Figure S3), preventing use of αKG at higher concentrations. However, the rate of turnover by GDH far exceeded the rate of turnover by AstC (>1,000-fold), as confirmed using ammonia as a positive control (data not shown). The concentration of αKG was therefore assumed to be non-limiting throughout the assays.

### Thermal Denaturation Studies

The AstC protein was used at 110 µM (5 mg/mL) and four duplications of the apo-AstC enzyme and four duplications the holo-enzyme (PLP/enzyme complex) were set up. Each experimental well contained 0.3 µL protein +0.3 µL 1/10 SYPRO dye (Sigma S5692) +19.4 µL Tris buffered saline solution.

### Modelling

All substrates were prepared by assigning protonation states using a neutral pH model as well as AM1BCC partial atomic charges [32] with QuacPac from OpenEye Scientific Software (Santa Fe, NM). Conformations for each ligand were enumerated using OpenEye’s Omega software. Hydrogens were added to the crystal structures using Reduce [33]. All docking calculations were performed with FRED 2.2.5 [34]. Two constraints were used during the docking process. The constraints were added such that an amine, ketone, or carboxylic acid had to be positioned within 3 Å of the Schiff base and that the ligand must form a hydrogen bond with Arg141 (i.e. the interaction needed to position the ligand for catalysis as revealed in our structure with bound SO). These constraints assure that all successfully docked poses will be consistent with our knowledge of this enzyme’s catalytic mechanism. Poses were scored and optimized with the Chemgauss3 scoring function [34] and the top 100 poses were ranked by a consensus of the following scoring functions: Chemgaus3, Piecewise Linear Potential (PLP), Shapegauss, Chemscore, OEChemscore, and Chemical Gaussian Overlay (CGO). The electrostatic potential for the protein active site was calculated using the Poisson-Boltzmann equation as implemented in the Zap toolkit from Openeye Scientific Software.

## Results

### Analysis of Purified AstC

The native, non-tagged AstC that was purified from *E. coli* was the apo-protein, with no bound PLP, as suggested by the lack of color of the purified protein, and confirmed by a UV-Vis absorbance spectrum, which showed no absorbance in the region of 320 to 500 nm (data not shown) which is characteristic of PLP containing proteins [35]. This was unexpected, as PLP is not limiting under the normal growth conditions used here (2x YT media) and it is generally bound covalently to a lysine as an aldimine for most PLP enzymes (for example [20]).

**Figure 2:**
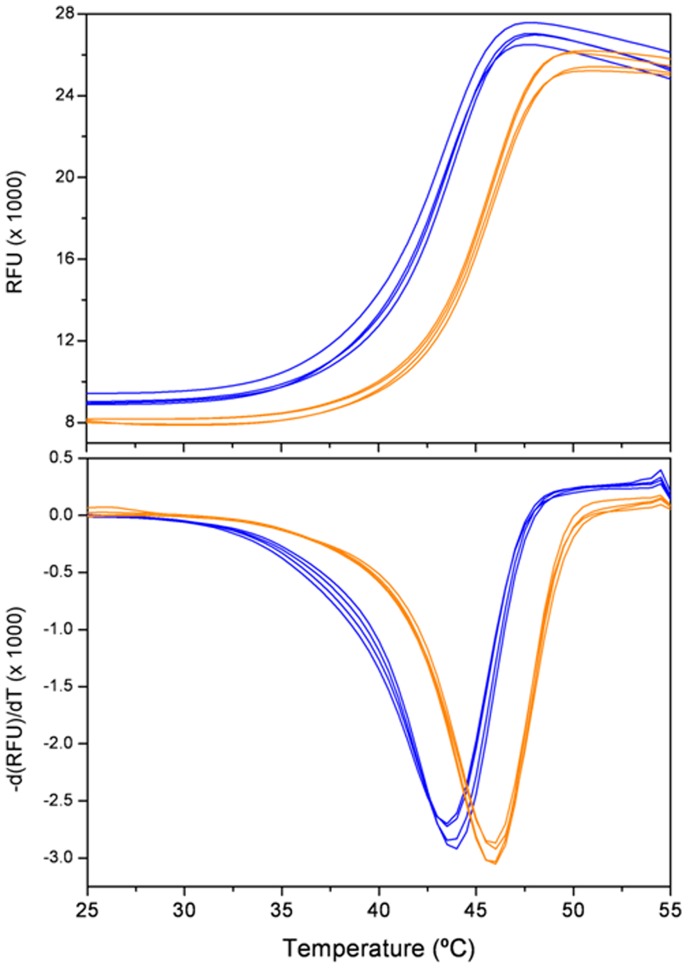
Differential Scanning Fluorimetry of AstC. Thermal denaturation curves obtained for AstC and AstC plus PLP cofactor. The blue curves are AstC, average *T*_h_ = 43.5°C; orange curves are AstC+PLP, average *T*_h_ = 46.0°C.

It was possible to reconstitute the holo-enzyme by the addition of PLP (Sigma P9255) to the apo-protein. The reconstituted enzyme was more heat stable than the apo-protein; holo-AstC and apo-AstC had apparent T_m_ values of 46°C and 43.5°C, respectively (Figure 2).

The activity of the reconstituted holo-AstC was tested against ORN, SO and AO (Table 2). The *K*_M_ data for ORN and AO are in reasonable agreement with those recorded by Ledwidge and Blanchard [19], who found *K*_M_ values of 4,500 and 150 µM for ORN and AO respectively (compared with 4,391 and 338 µM in this study). The *k*_cat_ values from the Ledwidge study were significantly lower than those described in this study; 4.7 s^−1^ for AO and 3.5 s^−1^ for ORN from this study compared to 0.61 s^−1^ for AO and 0.22 s^−1^ for ORN [19]. One possible explanation for this discrepancy is that the ArgD used in the earlier study was not fully occupied with PLP.

**Table 2:** Biochemical data on the activity of *E. coli* AstC, ArgD and *St*-ArgD.

Substrate		AstC^1^	ArgD^2^	ArgD^3^
**SO**	*k_cat_* (s^−1^)	3.0		
	*K*_M_ (µM)	284		
	*k*_cat_/*K*_M_ (M^−1^.s^−1^)	10,563		
**AO**	*k_cat_* (s^−1^)	4.7	1.6	0.61
	*K*_M_ (µM)	338	37	150
	*k*_cat_/*K*_M_ (M^−1^.s^−1^)	13,905	41,892	4040
**ORN**	*k_cat_* (s^−1^)	3.5	0.82	0.22
	*K*_M_ (µM)	4,391	640	4,500
	*k*_cat_/*K*_M_ (M^−1^.s^−1^)	797	1,281	49

1 *E. coli* from this study.

2 *Salmonella typhimurium* [20].

3 *E. coli* ArgD [19].

The more recent study from Rajaram and co-workers with *S. typhimurium* ArgD (*St*-ArgD) [20] also reported steady state kinetic values for ORN (*k*_cat_ = 0.82 s^−1^, *K*_M_ = 640 µM) and AO (*k*_cat_ = 1.6 s^−1^, *K*_M_ = 37 µM). Although the values for *K*_M_ obtained by Rajaram *et al* are somewhat lower than our own or those of Ledwidge and Blanchard, the order of substrate preference is the same in all three cases.

We also tested the activity of purified holo-AstC against its physiological substrate, SO. The *K*_M_ and *k*_cat_ values for SO (284 µM and 3 s^−1^, respectively) are slightly lower than those for AO, giving *k*_cat_/*K*_M_ values of 1×10^4^ M^−1^.s^−1^ and 1.4×10^4^ M^−1^.s^−1^ for SO and AO, respectively.

### Structures of Apo- and Holo-AstC

**Figure 3:**
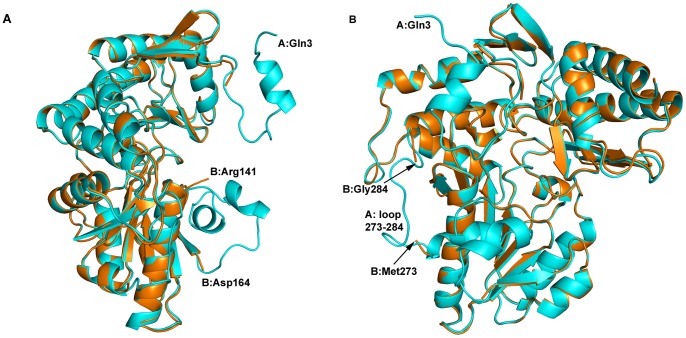
The apo-AstC structure. Figure 3A shows the superposition of apo-AstC protomer B (burnt orange) on protomer A (cyan) without PLP present in the structure. The N-terminus at the top right and the first 20 residues of protomer B are not modelled due to a lack of electron density for these residues. The other clear difference shown in 3A is loop 142–163, which is missing in protomer B (lower right). An approximate 90 degree rotation in 3B highlights the missing loop of residues 274–283 (lower left side of 3B).

We were able to obtain crystals of the apo-protein, although they diffracted to only medium resolution (2.75 Å). The structure of apo-AstC is shown in Figures 3A and 3B. The A and B protomers in the asymmetric unit differ substantially, with the A chain having electron density for almost the entire length of the molecule (Gln3 to Ser402) and the B chain starting at residue Ala21 and ending at Ser402. The B chain also has no electron density corresponding to two loops in the A chain from His142 to Ala163 and Val274 to Gly283, respectively. The two protomers do not form a dimer, rather dimers are formed through crystallographic symmetry for both, the A with another A protomer and the B with another B protomer. The loops missing from the B protomer form part of the dimer interface; analysis of the PISA [36] output shows that the A–A interface buries about 4763 Å^2^ whereas the B–B interface buries only 1906 Å^2^. Consistent with our spectrophotometric data, there was no electron density for the PLP co-factor in the crystal structures of protein obtained directly from purification.

**Figure 4:**
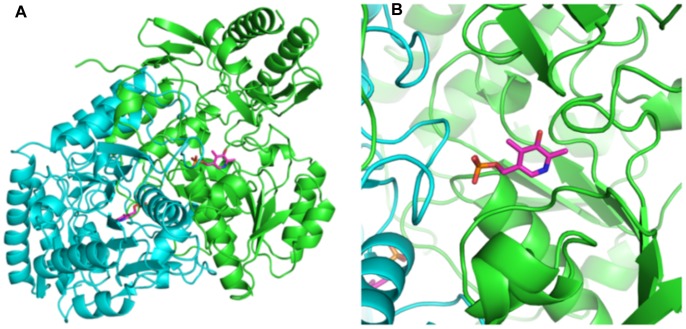
The native AstC structure with PLP. Figures 4A and 4B show the native dimer structure as a ribbon Cα trace with the PLP cofactor shown in a magenta colored stick representation. Figure 4B is a zoomed image showing how the loop of residues 274–284 comes in from the neighboring protomer to contact the PLP cofactor and form part of the active site of protomer A.

**Figure 5:**
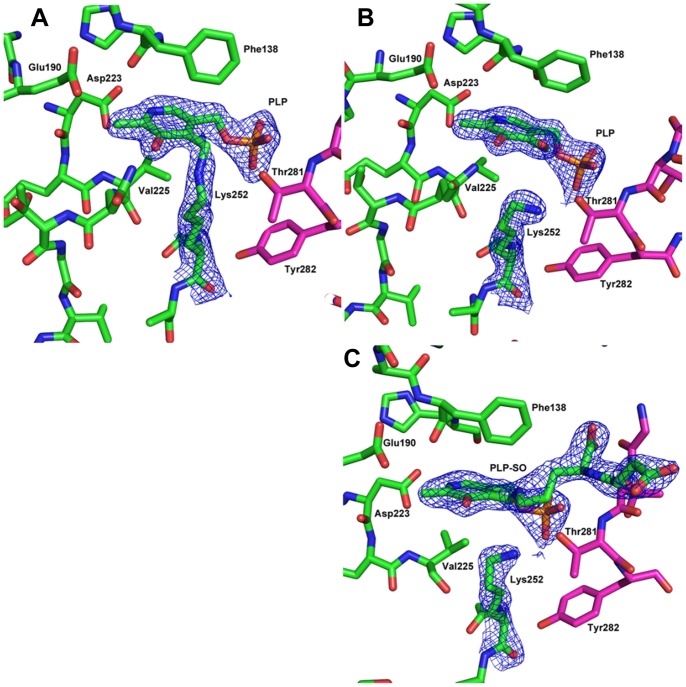
Structures of AstC prior and after reaction with substrates. Figure 5A shows the 2Fo-Fc density (blue mesh) for the PLP cofactor bound to Lys252 in a Schiff base. The B protomer is in green and the A protomer loop of 277–284 is shown in magenta. The electron density is set at 1.5 sigma. Figure 5B shows the density for PLP and Lys252 after the crystals were soaked with ORN. It is clear that the covalent bond between Lys252 and the PLP is broken and we have now modelled the PLP with an additional oxygen atom. Figure 5C shows the electron density for Lys252, PLP and the SO found in the SO soaked crystals. There is clean electron density that shows the SO covalently bound to the PLP and a break from Lys252. All density figures are 2Fo-Fc maps and were made with the ligands in the model.

Crystals of holo-AstC diffracted to a higher resolution than the apo-protein (2.20 Å). Figures 4A and 4B show a dimer of holo-AstC, with PLP clearly bound in the cofactor site at the dimer interface. There is unambiguous density for the cofactor, and there is continuous density between Lys252 and the PLP, indicating that the expected aldimine had formed between the lysine residue and the PLP (see Figure 5A). There are four independent protomers in the asymmetric unit in the C2 crystal form, two full dimers, each protomer being almost equally resolved. The PLP makes interactions with other residues in the binding site: the N1 of PLP is 2.6 to 2.8 Å from the Asp223 sidechain carboxylate, the O3 is 2.9 to 3.0 Å to the Gln226 sidechain, the PO_3_ oxygens are 2.9 to 3.1 Å to the Gly105 backbone nitrogen, 2.8 to 3.0 Å from the Ala106 backbone nitrogen, 2.9 to 3.1 Å from the Thr281 backbone nitrogen and 2.5 to 2.8 Å from the Thr281 sidechain oxygen (the distances vary slightly between the four protomers in the asymmetric unit). In this case, the A–B and C–D dimer interfaces are also effectively equal, with a PISA analysis showing that the average surface area buried is 4752 Å^2^ (4764 Å^2^ for A–B and 4740 Å^2^ for C–D). Dynamic light scattering indicates a hydrodynamic radius mean value 4.5 nm, indicative of a molecular weight of approximately 100 kDa (data not shown), suggesting that AstC is also a dimer in solution.

### Substrate/co-substrate Binding

To better understand the intricacies of the reaction, ORN, αKG and GLU were soaked into crystals of holo-AstC. The crystals soaked with GLU diffracted poorly (i.e. worse than 3 Å), these crystals were not of sufficient quality for further analyses. We found very little change in the structures obtained from αKG soaked crystals and were unable to detect electron density corresponding to the co-substrate.

We were also unable to find extra electron density for the ORN soaked crystals, but there was a distinct change in the active site, with the PLP-Schiff base bond to Lys252 clearly broken (see Figure 5B). This finding is consistent with the completion of the first half reaction (i.e. deamination of the substrate), producing an aldehyde and pyridoxamine. The lack of electron density for the substrate may be due to the high *K*_M_ of AstC for ORN (Table 2).

Electron density corresponding to the external aldimine enzyme:substrate complex was present in the structures obtained from crystals co-crystallized with SO. In these crystals the Lys252-PLP Schiff base was absent and continuous electron density between PLP and SO was observed (see Figure 5C). The SO makes remarkably few direct interactions with the protein and it seems that it is held in place mainly by the covalent bond to the cofactor. In addition to this, one of the carboxylates makes a salt bridge to Arg141, which varies in length from 2.8 to 3.1 Å. The other interactions are through water molecules to the protein (0 to 2 per protomer). There were also some minor changes outside of the cofactor binding site. The Phe138 side chain moves slightly, there is some movement in the loop consisting of residues 151–154 and the side chain of Tyr18 moves to accommodate the SO moiety. Apart from these differences, all three structures are remarkably similar.

**Figure 6:**
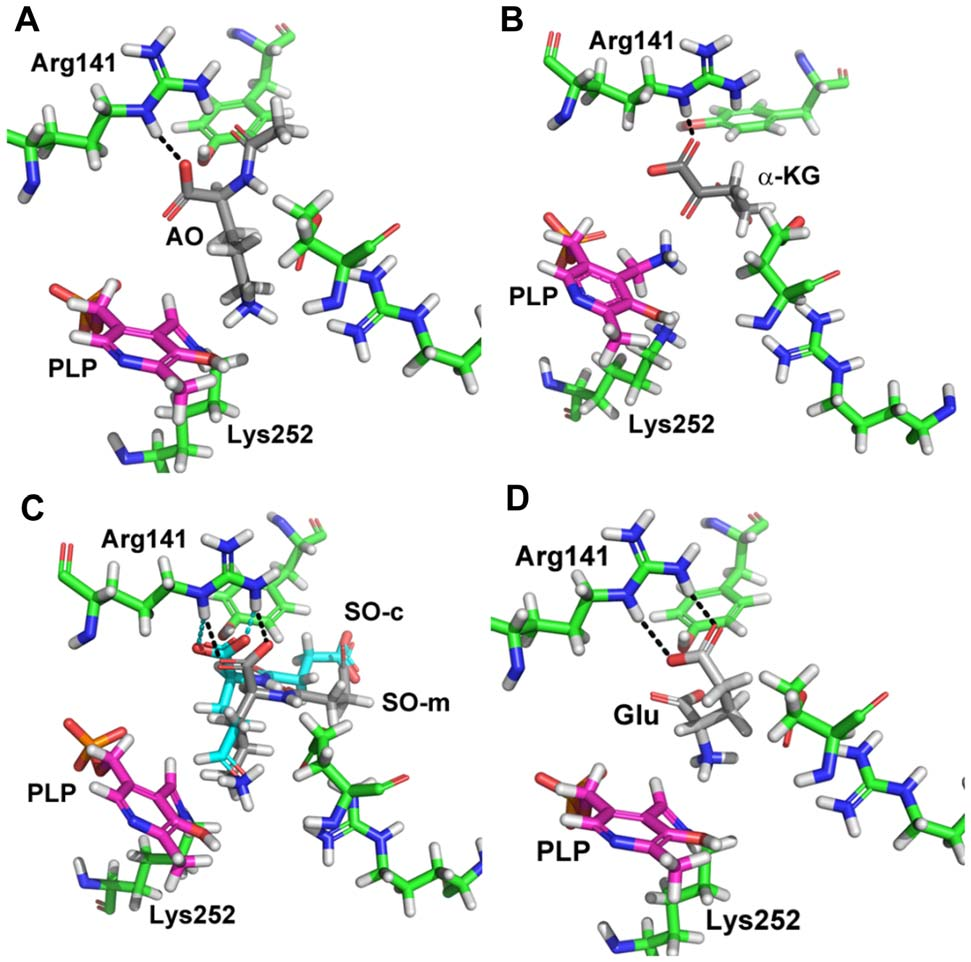
Modeled structures of AstC with substrates and products. 6A: Shown here is AO (grey) docked into the active site of SO transaminase. Pyridoxyl phosphate is shown in magenta covalently bound to Lys 252. Hydrogen bonds between Arg 141 and AO are shown in black. This is the highest scoring pose that was correctly oriented for catalysis (6 out of 100). 6B: Shown here is the top scoring conformation of α-ketoglutarate (grey) docked into SO transaminase with pyridoxamine phosphate (magenta). Hydrogen bonds with Arg 141 are shown in black. 6C: Shown here is the top-scoring pose of SO (grey, SO-m) docked into the active site of SO transaminase. Pyridoxyl phosphate is shown in magenta covalently bound to Lys 252. Hydrogen bonds between SO and Arg 141 are shown in black. In cyan is the product succinyl-glutamic semialdehyde (SO-c, derived from the crystal structure by replacing the covalently bound amine group with the appropriate aldehyde). Hydrogen bonds from this ligand to Arg 141 are shown in cyan. In this image the docked structure (grey) has a root-mean-square distance of 1.8Å relative to the crystal structure (cyan). 6D: Shown here is glutamate (grey) docked into the active site of SO transaminase. Pyridoxyl phosphate is shown in magenta covalently bound to Lys 252. Hydrogen bonds between Arg 141 and glutamate are shown in black. This is the highest scoring pose that was correctly oriented for catalysis (2 out of 100).

Using molecular docking we were able to predict plausible conformations for those ligands that were not observed in the X-ray structures. SO, ORN and AO were docked into the active site of the structure with PLP covalently bound to Lys252, while αKG was docked into the non-covalently bound pyridoxamine phosphate structure. By constraining the docking process to produce poses that form hydrogen bonds with Arg141 and place either an amine, a ketone, or a carboxylate functional group within 3 Å of the catalytic nitrogen, we were able to obtain reasonable docked conformations for all molecules. Furthermore, using this method, the crystallographic ligand (SO) was docked within 1.8 Å of the crystallographic conformation (Figure 6C). Additionally, for all but AO, the top ranking docked conformation was in an orientation consistent with the known catalytic mechanism. In the case of AO, the highest-ranking conformation consistent with the known mechanism was ranked 6^th^ (out of 100).

We see that αKG is positioned with the ketone carbon ready for attack by the amino nitrogen of pyridoxamine phosphate. Additionally, the carbonyl group is stabilized by interactions with Arg141 (Figure 6B). For the ornithine-derived substrates we see the same trend. The side chain amine is positioned proximally to the Schiff nitrogen of the covalently bound PLP. The backbone portion of ORN and its acylated derivatives are stabilized by interactions with Arg141 (Figure 6C). We also see for many docked conformations, Glu195 and Arg194 together serve to stabilize different charged groups near the Schiff base.

Although AstC can catalyze the reverse reaction (glutamate to α-ketoglutarate), in order to do so, GLU must bind to the PLP-bound state and interact with the aldimine. Upon docking GLU into this structure we find that the backbone carboxylate group interacts with Arg141 positioning the amino nitrogen for catalysis (figure 6D). It may at first be surprising to see that GLU and αKG do not interact in the same fashion, but this seems reasonable considering that they must bind to two different states of the enzyme for catalysis.

## Discussion

### AstC *vs.* ArgD

**Figure 7:**
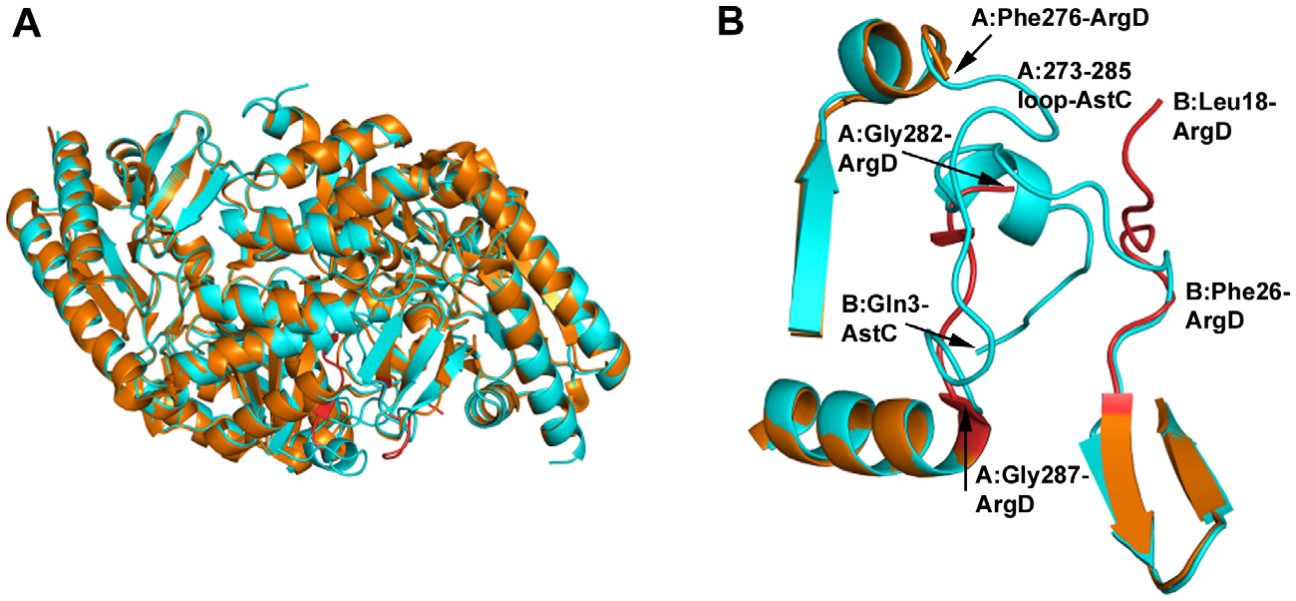
Comparison of AstC structure with ArgD from *S. typhimurium*. Figure 7A shows the structure of 2PB0 (in burnt orange) with a dimer of AstC overlayed (cyan). The structures are very similar with the major differences being in the N-terminus (highlighted in 7B) and the loop of 277–286. Figure 7B again has the 2PB0 structure in orange and AstC in cyan, but the differences in 2PB0 are highlighted in red. On the top right hand side we have the start of the 2PB0 B chain, Leu18, which does not come into alignment with AstC until residue Phe26. At the top we have the two structures aligned at residue Phe276 where the density falls off for 2PB0 and there is a gap to residue Gly282 (hidden in the helix of the N-terminus of AstC). The 2PB0 model for this part of the chain comes back into alignment with AstC just before the helix at residue Gly287 (bottom of figure).

A comparison of the AstC structure with the ArgD structures from *Samonella typhimurium* (PDB codes 2PB0 and 2PB2) shows that the overall fold is the same but with several discrete differences (Figure 7A). The start of the structural alignment (as performed in Coot [28]) is around Ala21/Phe23 (A or B chains, respectively), although there are several conserved residues prior to this in the sequence. 244 out of 405/406 residues in the sequence are identical, with Ser402 being the last residue seen in the AstC structure corresponding to residue 405 in 2PB2 (the last 4 residues of AstC, Arg-Gly-Ser-Ser, are not conserved, do not have electron density and are not modelled). Although the sequence identity is less than 50% for the 100 residues in the C-termini, the backbones of the structures essentially overlap in this region.

The *St*-ArgD structures are most similar to the B protomer of the apo-enzyme structure of AstC reported here, in that the N-terminal ∼23 amino acids of the structures are missing as are six residues in a loop from 277–282. These six residues are not modelled in three out of four independent *St*-ArgD monomers. The electron density for these residues is unambiguous in all of the PLP-bound structures of AstC and the A protomer of the PLP-free structure. In the AstC structures, the loop 277–282 ventures into the catalytic site of the neighboring protomer and makes important interactions to the cofactor (the distance from the Thr281 backbone nitrogen to an oxygen of PLP varies from 2.75 to 3.10 Å and the hydroxyl of Thr281 has a hydrogen bond of 2.5 to 3.0 Å to a phosphate oxygen). The equivalent *St*-ArgD Thr284 (in the one loop that exists in 2PB0) is also within hydrogen bonding distance to an oxygen of the PLP phosphate (2.54 Å). As this is part of the dimer interface, it is no surprise that the inferred dimer interfaces for *St*-ArgD are smaller than for AstC, with PISA giving 3600 Å^2^ for 2PB0 and 2744 Å^2^ for 2PB2 compared with 4752 Å^2^ for holo-AstC.

The structure factors deposited in the RCSB for 2PB0/2PB2 give electron density maps that clearly show that the PLP is not covalently bound to Lys255 *via* a Schiff base and that the PLP cofactors are not fully occupied in the pocket of ArgD (see Figure S4), in contrast to what is stated in the paper describing the structure [20]. This partial occupancy suggests that the recombinant *St*-ArgD was not fully saturated with PLP, and is consistent with the observation that AstC purified as an apo-protein, suggesting a low affinity of both ArgD and AstC for their PLP co-factor. These two observations lend credence to the suggestion that the earlier activity assay work of Ledwidge and Blanchard may have been affected by having limiting PLP in the protein sample. The lack of internal aldimine in the ArgD structures is similar to the structure of AstC after soaking with ORN, suggesting that the majority of *St*-ArgD had undergone a half reaction.

At the N-termini of the AstC and *St*-ArgD structures there are a number of differences (Figure 7B). The AstC structures start at residue Gln3 and come into alignment with 2PB2 at residue Ala21 (Ala24 in the A chain of 2PB2). In the B chain of 2PB2, the model of *St*-ArgD starts at residue Leu18 and comes into alignment with AstC at residue Phe26/Ile27 (23/24 in the AstC numbering). An interesting feature is that the A chain of AstC at residue Trp13 starts to overlap with the model for the 2PB2 structure at Ser283B. That is to say, Ser283-Thr284 of the B chain of 2PB2 roughly occupies the same space as Trp13-Met14 of the A chain of AstC, before the 2PB2 structure moves off between two beta-strands and then rejoins the AstC model for chain B at residue Gly287 (Gly284 in AstC). The reciprocal relationship is also true but with an extra residue, where part of the N-terminus of the AstC B chain is overlapping with the middle of the A chain of 2PB2. We believe this is due to the missing loop in the 2PB2/2PB0 structures, residues 277–282, which are clearly defined in the AstC structures but have inadequate electron density in the 2PB2/2PB0 structures for accurate structural modelling. In the one monomer of 2PB0 where there is sufficient electron density to model these residues, it follows the same path as in the AstC structures. But where these residues are missing, the 2PB2/2PB0 models deviate from this path and start the model from a completely different place (the N-terminus of the dimer partner in the AstC structures).

In the analysis of the electron density for structures 2PB0/2PB2, we found that there is clear density for a valine at position 298 in the sequence (see Figure S5), which is defined as an alanine in the sequence given by Rajaram et al and is an alanine in the Uniprot database. We expect that one of two scenarios is possible: there was a PCR error during the cloning of this gene from the genomic DNA used, or that there was a mutation at this position in the *S. typhimurium* genomic DNA. As this is a single base mutation (GCX for Ala, GUX for Val) either possibility is reasonable.

There are five ArgD structures currently available in the PDB from various organisms (*S. typhimurium*, *T. thermophilus*, *T. maritima*, *C. jejuni* and *A. aeolicus*, see Table 3). Superposing these with the AstC structure (using the SSM algorithm as implemented in Coot) shows that the N-termini do not align well until about position 20 (AstC numbering) and the first helix varies somewhat, but past the first helix, the alignment is very good with only small differences in the majority of the structures (there is some variation in 7 different turn/loop regions: Ala93 to Ala96, Asp122 to Ser129, Gly149 to Pro160, Thr266 to Gly284, Lys298 to Asn306, Thr321 to Glu331 and Asn344 to Lys351). There is also some variation in the C-termini. Of note is the region Thr266 to Gly284, which is the region that complements the binding site of the second protomer in the dimer. One structure, 3NX3, has this in an ‘open’ form and the 2PB2/2PB0 structures have this as disordered. Around the PLP site the residues are highly conserved, the only changes being Val225 is sometimes isoleucine and Ser104 is threonine in one instance (2ORD). The other changes nearby are: Asn109 to valine or isoleucine, His139 to serine and Tyr282 to phenylalanine, with these all being four or more Ångstroms away.

**Table 3:** Comparison of alignment of the AstD structures available in the PDB as of August, 2012 with the AstC structure determined in this study.

PDB	RMSD (Å)	# residues	# aa aligned	# gaps	Seq ID %
**2PB2**	0.6	378	373	1	60.6
**1VEF**	1.3	387	379	5	39.1
**2EH6**	1.2	375	370	5	43.8
**3NX3**	1.3	388	366	9	36.3
**2ORD**	1.1	393	390	6	39.2

From the biochemical data (Table 2), it appears that AstC and ArgD are biochemically similar, consistent with the ability of *astC* to complement an *argD* deletion [16]. Both have comparable steady state kinetic values, with a strong preference for the acylated ornithine derivatives AO (AstC and ArgD) and SO (for AstC, at least) over ORN itself.

When we look at the distribution of scores for the docked conformations, we find that we are able to reproduce the correct rank ordering of the ORN substrates relative to their experimental K_M_ values (Table 2, Figure S6, middle). Examination of the various terms in our scoring functions reveal that the increased affinity of the various ORN substrates results mostly from steric and desolvation effects (within the relative accuracy of the scoring function; Figure S6, bottom). This hypothesis is supported by the fact that the acetyl or succinyl moieties do not make any specific interactions with the active site, but rather are stabilized by positive electrostatic potential observed in this portion of the cavity (Figure S7). This hypothesis is sensible when considering the fact that a promiscuous enzyme binding different substrates in similar orientations would likely need to rely on non-specific interactions to accommodate all of its substrates. Having numerous specific interactions such hydrogen bond donors/acceptors in the active site would incur an energetic penalty when a given ligand cannot satisfy these interactions. Thus the most reasonable strategy for positioning different ligands in the same orientation is to rely on non-specific interactions to stabilize the enzyme-substrate complex. This is consistent with what we observe.

It seems that *E. coli* possesses two enzymes (AstC and ArgD) with interchangeable functionality and that the two have likely evolved from a common ancestral gene. Although the proteins are biochemically similar, they are components of differentially regulated transcriptional units, i.e. *astCADBE* and *argD*, and in these distinct regulatory contexts they perform different physiological roles. The I-A-D (innovation-amplification-divergence) model of evolution as proposed by Näsvall *et al* [37] proposes that a gene encoding a protein that has developed a promiscuous functionality may be duplicated, allowing both the physiological and promiscuous functionalities to become more efficient in their separate tasks [38]–[40]. Here, however, it is through divergence of the regulation of duplicate genes that each gene product has become adapted for specific physiological roles (anabolism and catabolism).

In conclusion, the *E. coli* AstC protein, involved in arginine catabolism, is a PLP containing transaminase that is structurally similar to the related protein ArgD, which is the transaminase enzyme used in arginine synthesis. The enzymes show similar kinetic values, although the current work does raise the possibility that previously published values of the ArgD kinetic parameters may be underestimated, due to a lack of saturating PLP in the enzyme samples. Docking studies show that AstC has a strong preference for acylated ornithine species over ORN, and suggest that the increase in specificity associated with acylation is caused by steric and desolvation effects rather than specific interactions between the substrate and enzyme.

## Supporting Information

Figure S1 **Crystals of AstC.** S1-A: Crystal of apo-AstC grown in 1.4 M sodium malonate pH 7, protein supplemented with silver bullet #62 (contains 0.2% D-fructose 1,6 diphosphate, 0.2% glycerol phosphate disodium salt, 0.2% L-O-phosphoserine, 0.2% O-phospho-L-tyrosine, 0.2% w/v phytic acid sodium salt, 0.02 M HEPES pH 6.8. Crystal is approximately 200 µm long in the longest dimension. S1-B: Crystals of AstC supplemented with PLP. These crystals grew in 1.17 M sodium malonate pH 7, 0.09 M Tris chloride pH 8, 0.2 M ethylammonium formate Crystals are 150 µm long in the longest dimension. S1-C: Crystals of AstC supplemented with PLP co-crystallized with succinyl ornithine. These crystals grew in 1.4 M ammonium sulfate, 0.1 M HEPES pH 7.5. The largest crystal is approximately 200 µm in the longest dimension.
(TIF)

Figure S2 Reaction scheme for succinyl ornithine starting from Boc-L-ornithine.
(TIF)

Figure S3 **Substrate inhibition of GDH.** Rate of GDH at varying (0–1300 mM) α-ketoglutarate concentrations in the presence of 12.5 µM NH3 and 2500 µM NAD+ and 0.1 U/µL GDH.
(TIF)

Figure S4 **The PLP density found for 2PB0.** Shows the negative density associated with the PLP-Lys255 bond in the 2PB0 structure as deposited in the Protein Data Bank. The orientation of 2PB0 is roughly equivalent to the AstC structure in Figure 4A. 2Fo-Fc density is shown in the blue chicken wire contoured at 1.0 sigma, the Fo-Fc density map is shown in the red chicken wire contoured at 3.0 sigma.
(TIF)

Figure S5 **Mutation found in the structure of 2PB0.** This figure shows the 2Fo–Fc density seen for Ala298 from 2PB0 and from 2PB2 (all four monomers show equivalent density). It is clear that this residue should be modelled as a valine, as the peaks for the Cγ1 and Cγ2 are 10–11 sigma in the difference maps.
(TIF)

Figure S6 **Plots of docked conformations using different scoring algorithms.** Top: Box-whisker plot of relative scores for correct (sidechain amine near catalytic nitrogen) versus backwards (back-bone amine near catalytic nitrogen) docked conformations using three different scoring functions: Shapegauss, PLP, and Chemgauss3. Middle: Box-whisker plots for the relative scores of docked conformations separated by substrate and by scoring function. Bottom: Box-whisker plot for the relative scores of docked conformations for different sub-terms of the Chemgauss3 scoring function.
(TIF)

Figure S7 **The electrostatic potential of the AstC binding pocket.** Shown here is the binding pocket of succinylornithine transaminase with the electrostatic potential of the apo pocket mapped to the molecular surface (colored from −5k_B_T in red to 5k_B_T in blue). The crystallographic ligand succinalglutamic semialdehyde is shown in cyan. The presence of large patches with highly positive potential can readily be seen.
(TIF)

Supporting Information S1 Supporting modelling information.
(DOCX)

## References

[1] Lu C-D (2006) Pathways and regulation of bacterial arginine metabolism and perspectives for obtaining arginine overproducing strains. Applied Microbiology and Biotechnology 70: 261–272 doi:10.1007/s00253-005-0308-z 16432742 10.1007/s00253-005-0308-z

[2] Tabor H, Tabor CW (1969) Formation of 1,4-Diaminobutane and of Spermidine by an Ornithine Auxotroph of Escherichia coli Grown on Limiting Ornithine or Arginine. Journal of Biological Chemistry 244: 2286–2292. 4891156

[3] Qian Z-G, Xia X-X, Lee SY (2009) Metabolic engineering of Escherichia coli for the production of putrescine: A four carbon diamine. Biotechnol Bioeng 104: 651–662 doi:10.1002/bit.22502 19714672 10.1002/bit.22502

[4] Tufvesson P, Lima-Ramos J, Jensen JS, Al-Haque N, Neto W, et al (2011) Process considerations for the asymmetric synthesis of chiral amines using transaminases. Biotechnology and Bioengineering 108: 1479–1493 doi:10.1002/bit.23154 21455931 10.1002/bit.23154

[5] Calogero S, Gardan R, Glaser P, Schweizer J, Rapoport G, et al (1994) RocR, a novel regulatory protein controlling arginine utilization in Bacillus subtilis, belongs to the NtrC/NifA family of transcriptional activators. Journal of Bacteriology 176: 1234–1241. 8113162 10.1128/jb.176.5.1234-1241.1994 PMC205184

[6] Gardan R, Rapoport G, Débarbouillé M (1995) Expression of the rocDEF Operon Involved in Arginine Catabolism in Bacillus subtilis. Journal of Molecular Biology 249: 843–856 doi:10.1006/jmbi.1995.0342 7540694 10.1006/jmbi.1995.0342

[7] Lüthi E, Baur H, Gamper M, Brunner F, Villeval D, et al (1990) The arc operon for anaerobic arginine catabolism in Pseudomonas aeruginosa contains an additional gene, arcD, encoding a membrane protein. Gene 87: 37–43 doi:10.1016/0378-1119(90)90493-B 2158926 10.1016/0378-1119(90)90493-b

[8] Bourdineaud JP, Heierli D, Gamper M, Verhoogt HJ, Driessen AJ, et al (1993) Characterization of the arcD arginine:ornithine exchanger of Pseudomonas aeruginosa. Localization in the cytoplasmic membrane and a topological model. Journal of Biological Chemistry 268: 5417–5424. 8449902

[9] Verhoogt HJ, Smit H, Abee T, Gamper M, Driessen AJ, et al (1992) arcD, the first gene of the arc operon for anaerobic arginine catabolism in Pseudomonas aeruginosa, encodes an arginine-ornithine exchanger. Journal of Bacteriology 174: 1568–1573. 1311296 10.1128/jb.174.5.1568-1573.1992 PMC206552

[10] Fan CL, Rodwell VW (1975) Physiological consequences of starvation in Pseudomonas putida: degradation of intracellular protein and loss of activity of the inducible enzymes of L-arginine catabolism. Journal of Bacteriology 124: 1302–1311. 1194237 10.1128/jb.124.3.1302-1311.1975 PMC236042

[11] Vanderbilt AS, Gaby NS, Rodwell VW (1975) Intermediates and enzymes between alpha-ketoarginine and gamma-guanidinobutyrate in the L-arginine catabolic pathway of Pseudomonas putida. Journal of Biological Chemistry 250: 5322–5329. 237915

[12] Tricot C, Stalon V, Legrain C (1991) Isolation and characterization of Pseudomonas putida mutants affected in arginine, ornithine and citrulline catabolism: function of the arginine oxidase and arginine succinyltransferase pathways. Journal of General Microbiology 137: 2911–2918 doi:10.1099/00221287-137-12-2911 1791443 10.1099/00221287-137-12-2911

[13] Itoh Y (1997) Cloning and characterization of the aru genes encoding enzymes of the catabolic arginine succinyltransferase pathway in Pseudomonas aeruginosa. Journal of Bacteriology 179: 7280–7290. 9393691 10.1128/jb.179.23.7280-7290.1997 PMC179677

[14] Stalon V, Mercenier A (1984) L-Arginine Utilization by Pseudomonas Species. Journal of General Microbiology 130: 69–76 doi:10.1099/00221287-130-1-69 6423769 10.1099/00221287-130-1-69

[15] Baumberg S, Harwood CR (1979) Carbon and nitrogen repression of arginine catabolic enzymes in Bacillus subtilis. Journal of Bacteriology 137: 189–196. 104957 10.1128/jb.137.1.189-196.1979 PMC218435

[16] Schneider BL, Kiupakis AK, Reitzer LJ (1998) Arginine Catabolism and the Arginine Succinyltransferase Pathway in Escherichia coli. Journal of Bacteriology 180: 4278–4286. 9696779 10.1128/jb.180.16.4278-4286.1998 PMC107427

[17] Kiupakis AK, Reitzer L (2002) ArgR-Independent Induction and ArgR-Dependent Superinduction of the astCADBE Operon in Escherichia coli. Journal of Bacteriology 184: 2940–2950 doi:10.1128/JB.184.11.2940-2950.2002 12003934 10.1128/JB.184.11.2940-2950.2002 PMC135064

[18] Fraley CD, Kim JH, McCann MP, Matin A (1998) The Escherichia coli Starvation Gene cstC Is Involved in Amino Acid Catabolism. Journal of Bacteriology 180: 4287–4290. 9696780 10.1128/jb.180.16.4287-4290.1998 PMC107428

[19] Ledwidge R, Blanchard JS (1999) The Dual Biosynthetic Capability of N-Acetylornithine Aminotransferase in Arginine and Lysine Biosynthesis. Biochemistry 38: 3019–3024 doi:10.1021/bi982574a 10074354 10.1021/bi982574a

[20] Rajaram V, Ratna Prasuna P, Savithri HS, Murthy MRN (2008) Structure of biosynthetic N-acetylornithine aminotransferase from Salmonella typhimurium: Studies on substrate specificity and inhibitor binding. Proteins 70: 429–441 doi:10.1002/prot.21567 17680699 10.1002/prot.21567

[21] Kim J, Copley SD (2007) Why Metabolic Enzymes Are Essential or Nonessential for Growth of Escherichia coli K12 on Glucose. Biochemistry 46: 12501–12511 doi:10.1021/bi7014629 17935357 10.1021/bi7014629

[22] Tiwari N, Woods L, Haley R, Kight A, Goforth R, et al (2010) Identification and characterization of native proteins of Escherichia coli BL-21 that display affinity towards Immobilized Metal Affinity Chromatography and Hydrophobic Interaction Chromatography Matrices. Protein Expression and Purification 70: 191–195 doi:10.1016/j.pep.2009.10.018 19887109 10.1016/j.pep.2009.10.018

[23] Newman J (2004) Novel buffer systems for macromolecular crystallization. Acta Crystallographica Section D: Biological Crystallography 60: 610–612. 14993709 10.1107/S0907444903029640

[24] Kabsch W (2010) XDS. Acta Crystallographica Section D Biological Crystallography 66: 125–132 doi:10.1107/S0907444909047337 20124692 10.1107/S0907444909047337 PMC2815665

[25] Evans P (2005) Scaling and assessment of data quality. Acta Crystallographica Section D Biological Crystallography 62: 72–82 doi:10.1107/S0907444905036693 16369096 10.1107/S0907444905036693

[26] Winn MD, Ballard CC, Cowtan KD, Dodson EJ, Emsley P, et al (2011) Overview of the CCP4 suite and current developments. Acta Crystallographica Section D Biological Crystallography 67: 235–242 doi:10.1107/S0907444910045749 21460441 10.1107/S0907444910045749 PMC3069738

[27] McCoy AJ, Grosse-Kunstleve RW, Adams PD, Winn MD, Storoni LC, et al (2007) Phaser crystallographic software. Journal of Applied Crystallography 40: 658–674 doi:10.1107/S0021889807021206 19461840 10.1107/S0021889807021206 PMC2483472

[28] Emsley P, Lohkamp B, Scott WG, Cowtan K (2010) Features and development of Coot. Acta Crystallographica Section D Biological Crystallography 66: 486–501 doi:10.1107/S0907444910007493 20383002 10.1107/S0907444910007493 PMC2852313

[29] Murshudov GN, Skubák P, Lebedev AA, Pannu NS, Steiner RA, et al (2011) REFMAC 5 for the refinement of macromolecular crystal structures. Acta Crystallographica Section D Biological Crystallography 67: 355–367 doi:10.1107/S0907444911001314 21460454 10.1107/S0907444911001314 PMC3069751

[30] Bergmeyer H-U, Beutler H-O (1985) Ammonia. Methods of Enzymatic Analysis. New York, NY: Academic Press, Vol. 8: 454–461.

[31] Chen SS, Engel PC (1975) Reversible modification of pig heart mitochondrial malate dehydrogenase by pyridoxal 5’-phosphate. Biochem J 151: 297–303. 175777 10.1042/bj1510297 PMC1172360

[32] Jakalian A, Jack DB, Bayly CI (2002) Fast, efficient generation of high-quality atomic charges. AM1-BCC model: II. Parameterization and validation. Journal of Computational Chemistry 23: 1623–1641 doi:10.1002/jcc.10128 12395429 10.1002/jcc.10128

[33] Word JM, Lovell SC, Richardson JS, Richardson DC (1999) Asparagine and glutamine: using hydrogen atom contacts in the choice of side-chain amide orientation. Journal of Molecular Biology 285: 1735–1747 doi:10.1006/jmbi.1998.2401 9917408 10.1006/jmbi.1998.2401

[34] McGann M (2011) FRED Pose Prediction and Virtual Screening Accuracy. Journal of Chemical Information and Modeling 51: 578–596 doi:10.1021/ci100436p 21323318 10.1021/ci100436p

[35] Metzler CM, Cahill A, Metzler DE (1980) Equilibriums and absorption spectra of Schiff bases. Journal of the American Chemical Society 102: 6075–6082 doi:10.1021/ja00539a017

[36] Krissinel E, Henrick K (2007) Inference of Macromolecular Assemblies from Crystalline State. Journal of Molecular Biology 372: 774–797 doi:10.1016/j.jmb.2007.05.022 17681537 10.1016/j.jmb.2007.05.022

[37] Nasvall J, Sun L, Roth JR, Andersson DI (2012) Real-Time Evolution of New Genes by Innovation, Amplification, and Divergence. Science 338: 384–387 doi:10.1126/science.1226521 23087246 10.1126/science.1226521 PMC4392837

[38] Khersonsky O, Tawfik DS (2010) Enzyme Promiscuity: A Mechanistic and Evolutionary Perspective. Annu Rev Biochem 79: 471–505 doi:10.1146/annurev-biochem-030409-143718 20235827 10.1146/annurev-biochem-030409-143718

[39] Copley S (2003) Enzymes with extra talents: moonlighting functions and catalytic promiscuity. Curr Opin Chem Biol 7: 265–272. 12714060 10.1016/s1367-5931(03)00032-2

[40] Tracewell CA, Arnold FH (2009) Directed enzyme evolution: climbing fitness peaks one amino acid at a time. Current Opinion in Chemical Biology 13: 3–9 doi:10.1016/j.cbpa.2009.01.017 19249235 10.1016/j.cbpa.2009.01.017 PMC2703427

