# Supplementary figures and images for: Determination of the Structure of the Catabolic N-Succinylornithine Transaminase (AstC) from Escherichia coli
Source: PLoS One. 2013 Mar 6;8(3):e58298. doi: 10.1371/journal.pone.0058298 (PMC3590144; doi:10.1371/journal.pone.0058298)

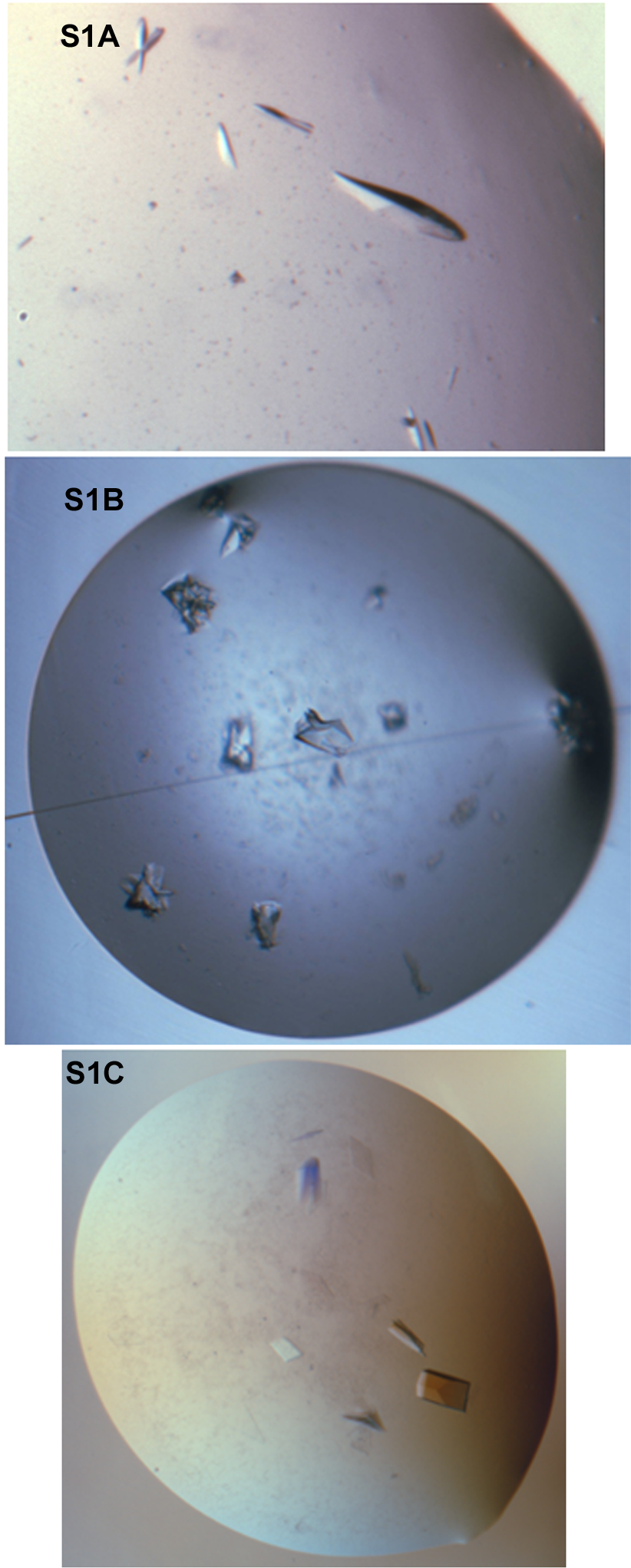

Supplement: Figure S1 — Crystals of AstC. S1-A: Crystal of apo-AstC grown in 1.4 M sodium malonate pH 7, protein supplemented with silver bullet #62 (contains 0.2% D-fructose 1,6 diphosphate, 0.2% glycerol phosphate disodium salt, 0.2% L-O-phosphoserine, 0.2% O-phospho-L-tyrosine, 0.2% w/v phytic acid sodium salt, 0.02 M HEPES pH 6.8. Crystal is approximately 200 µm long in the longest dimension. S1-B: Crystals of AstC supplemented with PLP. These crystals grew in 1.17 M sodium malonate pH 7, 0.09 M Tris chloride pH 8, 0.2 M ethylammonium formate Crystals are 150 µm long in the longest dimension. S1-C: Crystals of AstC supplemented with PLP co-crystallized with succinyl ornithine. These crystals grew in 1.4 M ammonium sulfate, 0.1 M HEPES pH 7.5. The largest crystal is approximately 200 µm in the longest dimension. (TIF) [file pone.0058298.s001.tif]

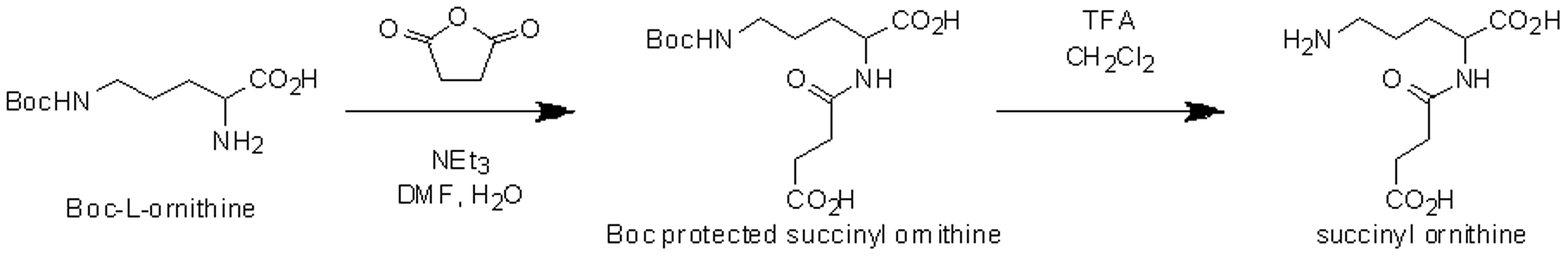

Supplement: Figure S2 — Reaction scheme for succinyl ornithine starting from Boc-L-ornithine. (TIF) [file pone.0058298.s002.tif]

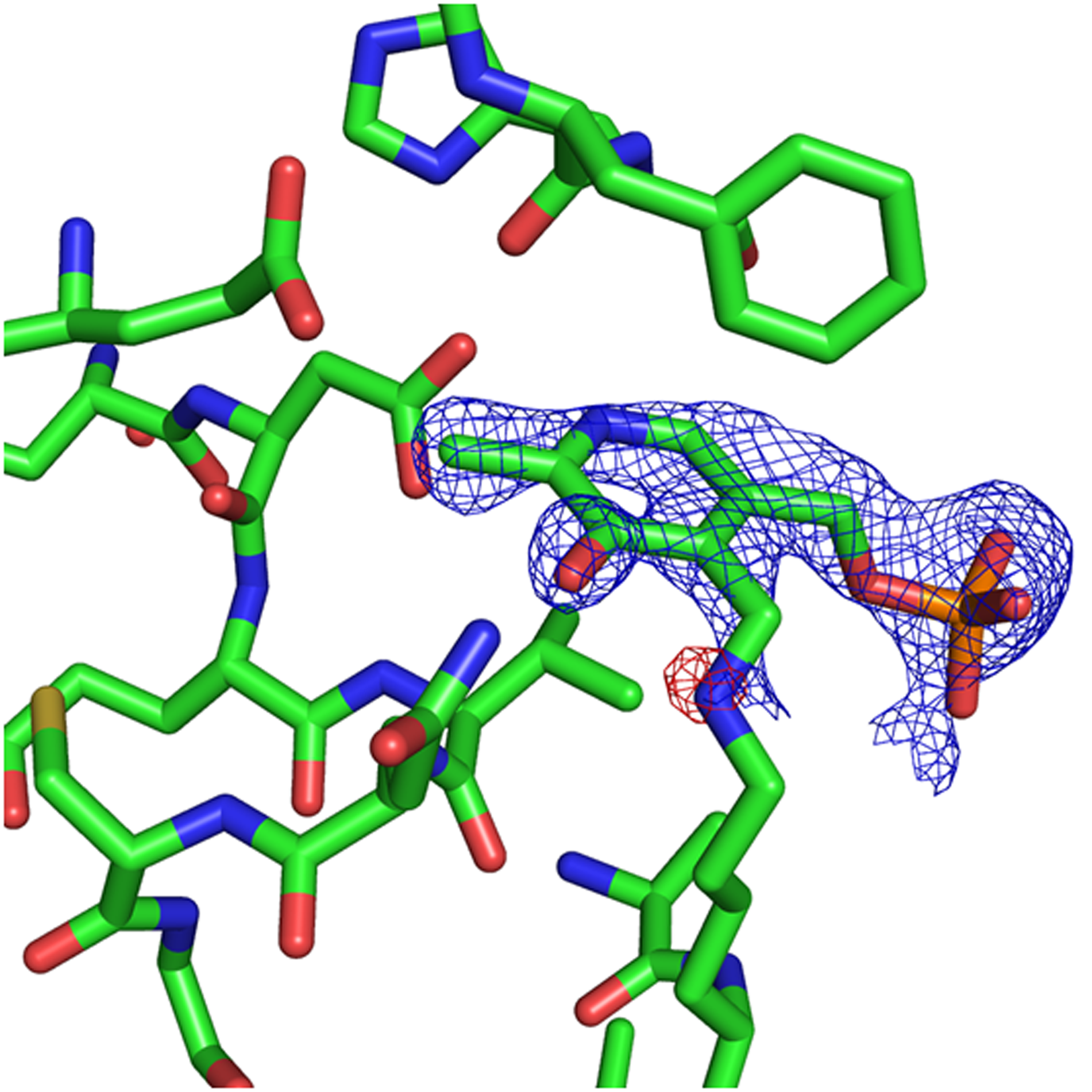

Supplement: Figure S4 — The PLP density found for 2PB0. Shows the negative density associated with the PLP-Lys255 bond in the 2PB0 structure as deposited in the Protein Data Bank. The orientation of 2PB0 is roughly equivalent to the AstC structure in Figure 4A. 2Fo-Fc density is shown in the blue chicken wire contoured at 1.0 sigma, the Fo-Fc density map is shown in the red chicken wire contoured at 3.0 sigma. (TIF) [file pone.0058298.s004.tif]

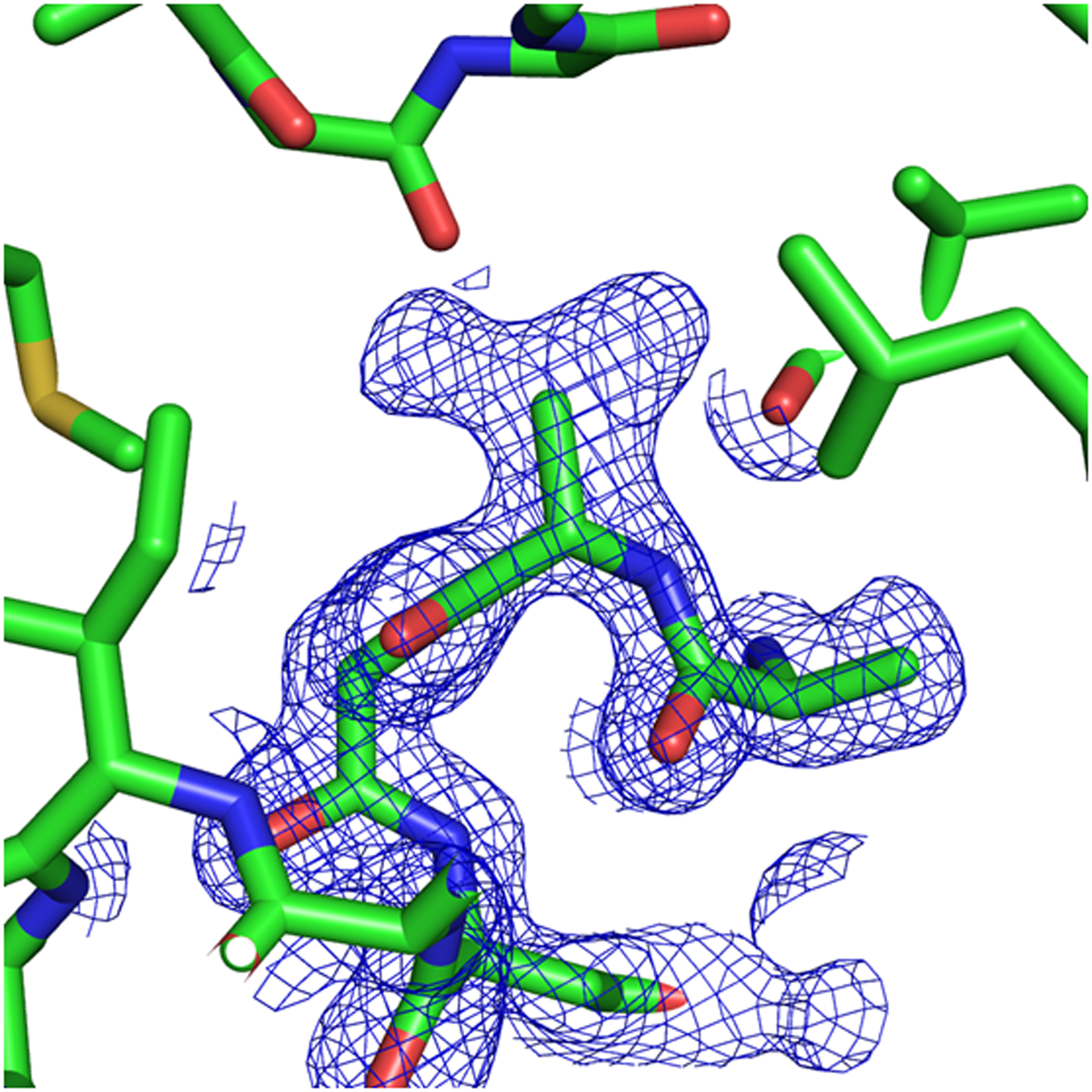

Supplement: Figure S5 — Mutation found in the structure of 2PB0. This figure shows the 2Fo–Fc density seen for Ala298 from 2PB0 and from 2PB2 (all four monomers show equivalent density). It is clear that this residue should be modelled as a valine, as the peaks for the Cγ1 and Cγ2 are 10–11 sigma in the difference maps. (TIF) [file pone.0058298.s005.tif]

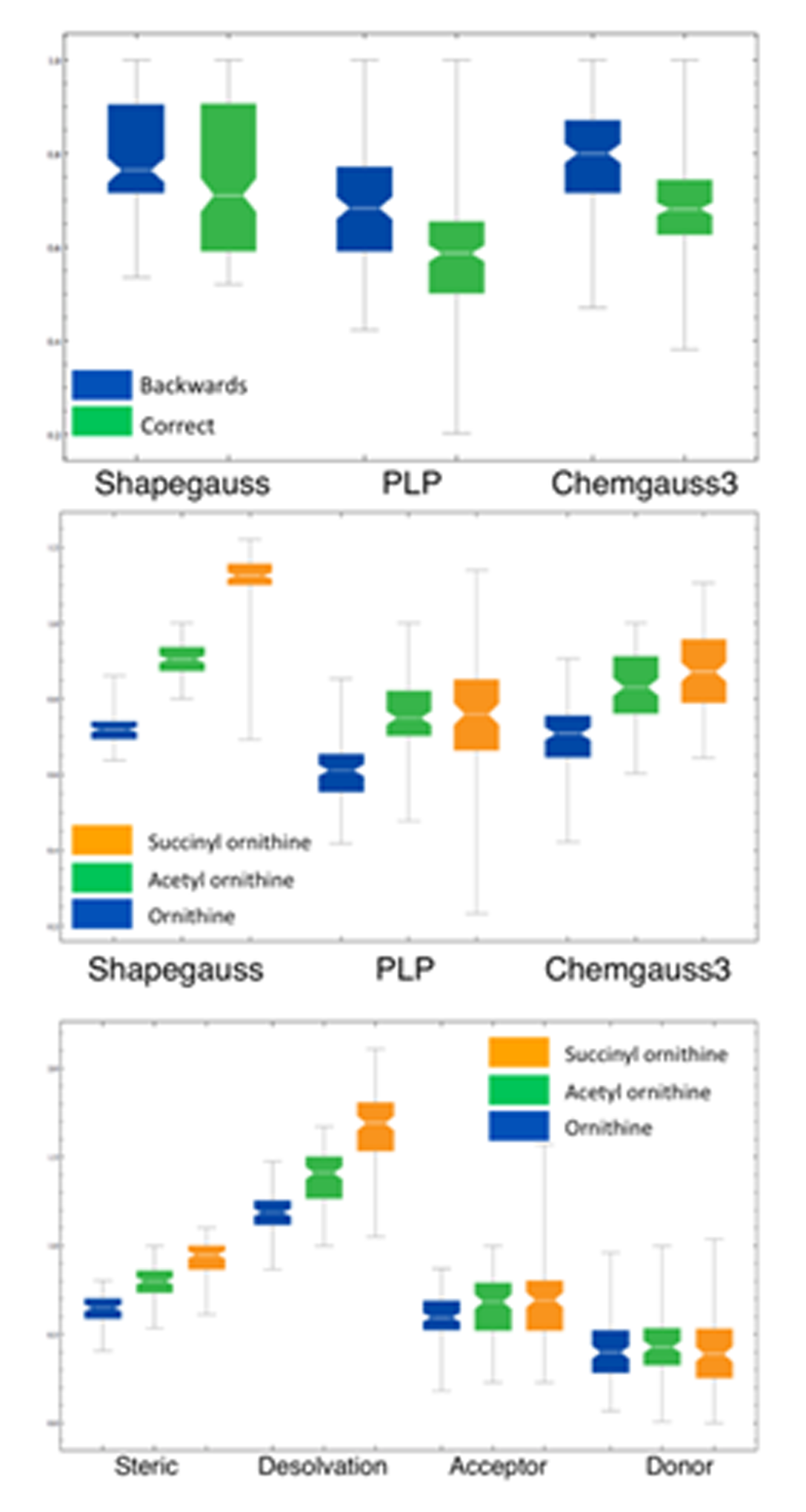

Supplement: Figure S6 — Plots of docked conformations using different scoring algorithms. Top: Box-whisker plot of relative scores for correct (sidechain amine near catalytic nitrogen) versus backwards (back-bone amine near catalytic nitrogen) docked conformations using three different scoring functions: Shapegauss, PLP, and Chemgauss3. Middle: Box-whisker plots for the relative scores of docked conformations separated by substrate and by scoring function. Bottom: Box-whisker plot for the relative scores of docked conformations for different sub-terms of the Chemgauss3 scoring function. (TIF) [file pone.0058298.s006.tif]

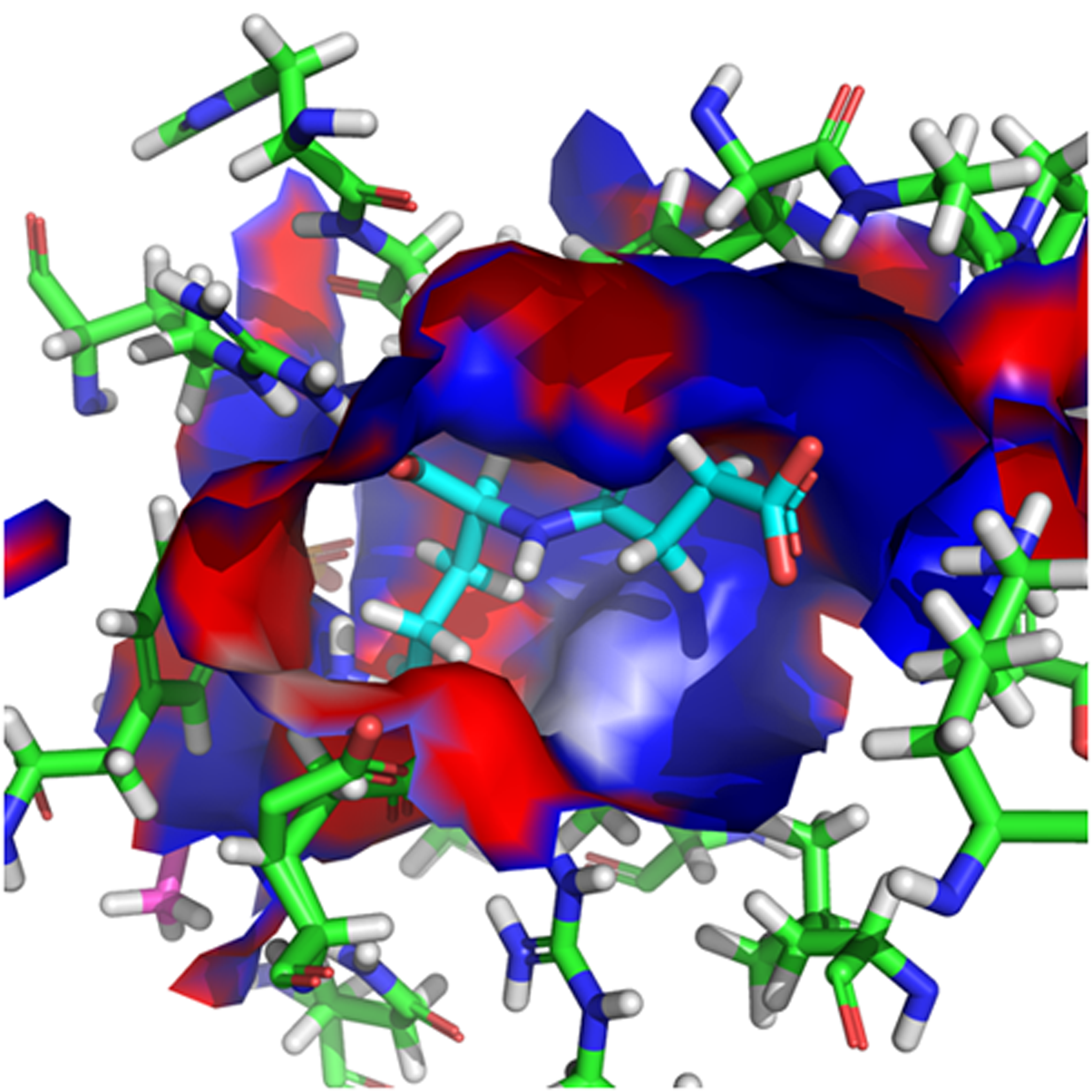

Supplement: Figure S7 — The electrostatic potential of the AstC binding pocket. Shown here is the binding pocket of succinylornithine transaminase with the electrostatic potential of the apo pocket mapped to the molecular surface (colored from −5kBT in red to 5kBT in blue). The crystallographic ligand succinalglutamic semialdehyde is shown in cyan. The presence of large patches with highly positive potential can readily be seen. (TIF) [file pone.0058298.s007.tif]
