# Supplementary material for: Determination of the Structure of the Catabolic N-Succinylornithine Transaminase (AstC) from Escherichia coli
Source: PLoS One. 2013 Mar 6;8(3):e58298. doi: 10.1371/journal.pone.0058298 (PMC3590144; doi:10.1371/journal.pone.0058298)
Supplement: Supporting Information S1 — Supporting modelling information. (DOCX) [file pone.0058298.s008.docx]

Supporting modelling information:

We realized that one potential complication with docking substrates into a trans-aminase is the ability of the docking algorithm and scoring function to distinguish between binding modes with the sidechain nitrogen in the binding pocket as opposed to the backbone nitrogen in the binding pocket (indeed some PLP-dependent transaminases attack the backbone nitrogen of their amino acid ligands) (reference S1). Although we applied constraints to ensure that an electronegative atom is proximal to the catalytic nitrogen, we did not force the ligands to bind in the correct orientation. Even though using a consensus scoring method produced a correctly oriented conformation as the best pose for all ligands except acetylornithine, we can see from the energy distribution of correct versus backwards binding that the conformations are nearly iso-energetic (from the point of view of the scoring functions used) (Figure S6 top). It is difficult to say if this is representative of a real physical difficulty in binding substrates in the correct orientation or just an artifact of the limited resolution of the scoring functions used.

Although scoring functions used in docking algorithms frequently show poor correlation to binding free energies (reference S2), we were nonetheless able to reproduce the correct ordering of affinities for the ornithine-derivatives with a number of scoring functions (Figure S6 middle). An examination of the sub-terms in the Chemgauss3 scoring function reveals that this effect may be simply a result of increased ligand surface area and desolvation of the active site rather than specific interactions between the protein and ligand (Figure S6 bottom). This is further supported by the fact that the succinate moiety of succinylornithine does not appear to be making any specific interactions in the crystal structure, but rather presents more polar surface area to the hydrophilic pocket. Visualizing the electrostatic potential in the pocket we can see that the field is quite positive, indicating a potential stabilizing effect for ligands with more negative charge (such as succinylornithine) (Figure S7).

Supporting references:

S1. Hammes, G. G. (2002) Multiple Conformational Changes in Enzyme Catalysis†. *Biochemistry* **41**, 8221–8228

S2. Warren, G. L., Andrews, C. W., Capelli, A.-M., Clarke, B., LaLonde, J., Lambert, M. H., Lindvall, M., Nevins, N., Semus, S. F., Senger, S., Tedesco, G., Wall, I. D., Woolven, J. M., Peishoff, C. E., and Head, M. S. (2005) A Critical Assessment of Docking Programs and Scoring Functions. *J. Med. Chem.* **49**, 5912–5931
